# Direct endothelial ENaC activation mitigates vasculopathy induced by SARS-CoV2 spike protein

**DOI:** 10.3389/fimmu.2023.1241448

**Published:** 2023-08-10

**Authors:** Maritza J. Romero, Qian Yue, Bhupesh Singla, Jürg Hamacher, Supriya Sridhar, Auriel S. Moseley, Chang Song, Mobarak A. Mraheil, Bernhard Fischer, Markus Zeitlinger, Trinad Chakraborty, David Fulton, Lin Gan, Brian H. Annex, Gabor Csanyi, Douglas C. Eaton, Rudolf Lucas

**Affiliations:** ^1^ Vascular Biology Center, Medical College of Georgia at Augusta University, Augusta, GA, United States; ^2^ Department of Pharmacology and Toxicology, Medical College of Georgia at Augusta University, Augusta, GA, United States; ^3^ Department of Medicine, School of Medicine, Emory University, Atlanta, GA, United States; ^4^ Pneumology, Clinic for General Internal Medicine, Lindenhofspital, Bern, Switzerland; ^5^ Lungen-und Atmungsstiftung, Bern, Switzerland; ^6^ Medical Clinic V—Pneumology, Allergology, Intensive Care Medicine, and Environmental Medicine, Faculty of Medicine, Saarland University, University Medical Centre of the Saarland, Homburg, Germany; ^7^ Institute for Medical Microbiology, German Centre for Infection Giessen-Marburg-Langen Site, Faculty of Medicine, Justus-Liebig University, Giessen, Germany; ^8^ Apeptico GmbH, Research and Development, Vienna, Austria; ^9^ Department of Clinical Pharmacology, Medical University of Vienna, Vienna, Austria; ^10^ Department of Neuroscience and Regenerative Medicine, Medical College of Georgia at Augusta University, Augusta, GA, United States; ^11^ Department of Medicine, Medical College of Georgia at Augusta University, Augusta, GA, United States; ^12^ Division of Pulmonary and Critical Care Medicine, Medical College of Georgia at Augusta University, Augusta, GA, United States

**Keywords:** Epithelial sodium channel (ENaC), SARS-CoV2 spike protein, receptor binding domain (RBD), human ACE-2, endothelial dysfunction, NADPH oxidase 2 (NOX2), tissue factor

## Abstract

**Introduction:**

Although both COVID-19 and non-COVID-19 ARDS can be accompanied by significantly increased levels of circulating cytokines, the former significantly differs from the latter by its higher vasculopathy, characterized by increased oxidative stress and coagulopathy in lung capillaries. This points towards the existence of SARS-CoV2-specific factors and mechanisms that can sensitize the endothelium towards becoming dysfunctional. Although the virus is rarely detected within endothelial cells or in the circulation, the S1 subunit of its spike protein, which contains the receptor binding domain (RBD) for human ACE2 (hACE2), can be detected in plasma from COVID-19 patients and its levels correlate with disease severity. It remains obscure how the SARS-CoV2 RBD exerts its deleterious actions in lung endothelium and whether there are mechanisms to mitigate this.

**Methods:**

In this study, we use a combination of *in vitro* studies in RBD-treated human lung microvascular endothelial cells (HL-MVEC), including electrophysiology, barrier function, oxidative stress and human ACE2 (hACE2) surface protein expression measurements with *in vivo* studies in transgenic mice globally expressing human ACE2 and injected with RBD.

**Results:**

We show that SARS-CoV2 RBD impairs endothelial ENaC activity, reduces surface hACE2 expression and increases reactive oxygen species (ROS) and tissue factor (TF) generation in monolayers of HL-MVEC, as such promoting barrier dysfunction and coagulopathy. The TNF-derived TIP peptide (a.k.a. solnatide, AP301) -which directly activates ENaC upon binding to its a subunit- can override RBD-induced impairment of ENaC function and hACE2 expression, mitigates ROS and TF generation and restores barrier function in HL-MVEC monolayers. In correlation with the increased mortality observed in COVID-19 patients co-infected with S. pneumoniae, compared to subjects solely infected with SARS-CoV2, we observe that prior intraperitoneal RBD treatment in transgenic mice globally expressing hACE2 significantly increases fibrin deposition and capillary leak upon intratracheal instillation of S. pneumoniae and that this is mitigated by TIP peptide treatment.

## Introduction

COVID-19 is a viral respiratory illness caused by the single stranded RNA virus, SARS-CoV2. The spike protein of SARS-CoV2 represents the main mediator of viral interaction with mammalian cells and has been the main target for vaccine development. The much higher transmissibility of SARS-CoV2 compared to SARS-CoV can be at least partially explained by the much higher affinity of its main receptor -human ACE-2- for the viral receptor binding domain (RBD), liberated by cleavage of an 8-residue sequence between the S1 from the S2 subunits of the spike protein by furin (1, 2). Disruption of the alveolar epithelial barrier results in pneumonia, but additional compromise of the endothelial barrier can lead to ARDS (3), which develops in about 20% of COVID-19 patients and is the main cause of mortality (4).

A significantly increased level of circulating pro-inflammatory cytokines can be detected in both COVID and non-COVID ARDS. Yet, COVID-19 pneumonia has a significantly higher vasculopathy as compared to other viral or bacterial pneumonia (5–12). SARS-CoV2-induced oxidative stress in lung capillaries is of particular importance for endothelial dysfunction in COVID-19, since it not only affects barrier function directly, but also indirectly, by inducing tissue factor generation and subsequent coagulopathy (5, 13). These findings point towards the existence of SARS-CoV2-mediated factors and mechanisms that can sensitize the endothelium towards barrier dysfunction in COVID-19. This can be especially relevant in the presence of pneumococcal co-infections, since concomitant SARS-CoV-2 infection and invasive pneumococcal disease (IPD) is associated with 7-fold higher risk of death, in comparison to IPD alone (14). These findings suggest that interactions between SARS-CoV-2 or its components and *S. pneumoniae* may aggravate ARDS (15). Although the virus is rarely detected within capillary endothelial cells or in the blood circulation (16), the S1 subunit of the spike protein, which contains the receptor binding domain (RBD) for human ACE2, can be detected in plasma from COVID-19 patients and its levels correlate with disease severity (17). Moreover, the SARS-CoV2 S1 subunit was shown by others and our group to induce barrier dysfunction in human lung microvascular endothelial cell (HL-MVEC) monolayers and in mice (18–20).

In this study, we investigate the role of oxidative stress in SARS-CoV2 RBD-induced barrier dysfunction in HL-MVEC monolayers. We moreover evaluate whether the TNF-derived TIP peptide (21–23) (a.k.a. solnatide, AP301), which activates endothelial ENaC and preserves barrier function in HL-MVEC or mice treated with bacterial toxins (24, 25), can also inhibit actions of the SARS-CoV2 spike protein in lung capillaries. We moreover assess whether prior intraperitoneal RBD injection can increase capillary leak in global human ACE2 transgenic mice subsequently infected with pneumococci. Our results demonstrate that RBD treatment inhibits ENaC activity, decreases hACE2 surface expression and increases ROS and tissue factor generation in HL-MVEC, as such causing direct and indirect barrier dysfunction. The TIP peptide, which binds to the α subunit of ENaC, can restore ENaC activity, hACE2 expression and barrier function in HL-MVEC in the presence of RBD, at least partially by reducing ROS generation. Our results indicate that SARS-CoV2 RBD represents a sensitizing factor for vasculopathy and coagulopathy in COVID-19 and that this is especially relevant for capillary leak during SARS-CoV2/pneumococcal co-infections. As such, strategies activating endothelial ENaC may hold promise to treat COVID-19-associated vasculopathy.

## Materials and methods

### Reagents

TIP peptide (a.k.a. Solnatide, AP-301), is a 17 amino acid cyclic synthetic peptide and a direct ENaC activator with the sequence CGQRETPEGAEAKPWYC and is provided by BCN, Barcelona, Spain. NADPH oxidase 2 inhibitory peptide gp91dstat is purchased from Anaspec (Freemont, CA, USA). Recombinant SARS-CoV2 spike protein RBD is purchased from Raybiotech (Peachtree Corners, GA, USA). Recombinant full length Spike protein is purchased from Creative Diagnostics (Shirley, NY, USA). Rabbit-anti-human ACE-2 polyclonal antibody and the human ACE-2 (CT) antibody blocking peptide are from PromoKine (PromoCell, Cat#: PK-AB718-3227, Heidelberg, Germany).

### Cell culture

Human lung microvascular endothelial cells (HL-MVEC), cell medium and endothelial cell growth supplement were purchased from Lonza (East Rutherford, NJ). Cells were cultured in humidified 5% CO_2_ at 37°C using the appropriate culture medium.

### Animals

Mice are housed in accordance with the National Institutes of Health (NIH) guidelines in the AAALAC-accredited experimental animal facility at Augusta University in a controlled environment. All mouse studies described in this study are approved by the Institutional Animal Care and Use Committee at Augusta University. Global humanized ACE-2 mice, in which mouse ACE2 has been globally replaced with human ACE2 (global hACE-2 knock in mice) have been generated using CRISPR-Cas9 in the Transgenic and Genome Editing Core facility at Augusta University. These mice express human ACE-2 in several cell types, including pulmonary endothelial cells. All mice are genotyped by PCR amplification of tail DNA. Every effort is made to minimize animal suffering and reduce the number of animals used.

### Intratracheal *S. pneumoniae* instillation

24h post i.p. RBD injection (500 μg/kg), i.t. instillation of 10^6^ CFU of D39 *S. pneumoniae* (26) (Institute of Medical Microbiology, Justus-Liebig University, Giessen, Germany) is performed. After sedation of the mice with i.p. ketamine (100 mg/kg) and xylazine (20 mg/kg) solution in 0.9% normal saline, animals are placed in an intubation platform, the tongue is carefully grasped with a curved blunt-ended forceps in an upward and leftward position to gain visualization of the larynx, and a fiber-optic illuminator is positioned over the trachea to trans-illuminate the tracheal opening. Mice are intubated using a 20-gauge catheter under a Zoom stereo microscope. A volume of 20-30 μl, containing either saline (vehicle), 10^6^ CFU of D39 *S. pneumoniae* or 10^6^ CFU of D39 *S. pneumoniae* with TIP peptide (2.5 mg/kg) is instilled. The catheter is removed, and the mice are maintained in the same position on the intubating platform for at least 30 s. Then, mice are removed from the platform and placed on a heating pad for a 3-4h recovery period. Animals are returned to isolated cages in the respective animal facilities for 24 h.

### Tissue harvesting

Tissue collection is performed in mice deeply anesthetized by i.p. injection of a ketamine (70-80 mg/kg) and xylazine (2-4 mg/kg) cocktail in sterile PBS at 24h post *S. pneumoniae* instillation. Following thoracotomy, lungs are removed and used for Evans Blue Dye extraction and immunohistology. Final euthanasia is guaranteed by exsanguination and organ removal.

### ROS generation in HL-MVEC

HL-MVEC are plated on coverslips inserted into wells of a 24-well plate. The next day, cells are treated for 2h with either vehicle (ctrl) or RBD (5 μg/ml, 2h), in the presence or absence of TIP peptide (50 μg/ml), the latter of which is applied either 30 min before or 30 min after RBD. Five min before the end of the above-mentioned treatments, CM-H2DCFDA solution (5 µM, Thermo Fisher, Cat#C6827) in serum-free media is added and cells are incubated for 15-30 min. Vehicle-treated cells without incubation with CM-H2DCFDA solution are taken as negative control (No H2DCFDA control). Coverslips with cells are washed twice with PBS, mounted on slides with DAPI-containing mounting media, and immediately imaged using a Zeiss 780 confocal microscope. Images of five randomly selected microscopic fields are captured (excitation/emission: 490 nm/525 nm) and fluorescence intensity is quantified using the Image-Pro Plus software (Media Cybernetics, Bethesda, MD).

### Human ACE-2 surface expression in HL-MVEC

We use cell surface biotinylation to determine if RBD (2 μg/ml) reduces surface hACE-2 expression in commercially available HL-MVEC and we investigate whether TIP peptide can prevent a RBD-induced reduction. Confluent cells are grown on permeable supports and washed three times with cold PBS buffer before adding 0.5 mg/ml sulfo-SS-biotin (Pierce) in borate buffer (85 mm NaCl, 4 mm KCl, 15 mmNa_2_B_4_O_7_, pH 9.0) to the apical surface while the basolateral compartment was exposed to media containing 5% (v/v) fetal calf serum. The experiment is performed at 4°C with gentle agitation for 15 min, and the procedure is repeated. Labeling is stopped at time zero by adding 5% fetal calf serum in endothelial cell medium. One filter sample from each group is scraped and lysed at times 0, 30 min, 1h, 2h and 4 h and 24h. Cells are extensively washed in PBS buffer, harvested, and lysed in buffer B (PBS with 0.1% SDS, 1% Nonidet P-40, and 0.5% sodium deoxycholate) containing protease inhibitors. Cellular debris is removed by centrifugation (1200 × *g*, 5 min). Biotin-labeled proteins are precipitated by incubating with prewashed streptavidin coupled to magnetic beads for 18 h at 4 °C with gentle agitation. The beads are collected with a magnetic stand and removed and the supernatant saved for analysis. 300μl of Buffer B is added to the tube and gently mixed. Beads are then collected and supernatant is discarded. This washing step is repeated twice. The biotin-streptavidin complex is then lysed by boiling in buffer containing 100 mm dithiothreitol and 5% SDS. Beads are magnetically separated and supernatant containing cell surface proteins is saved. The proteins are then separated in 7.5% SDS-PAGE, transferred to nitrocellulose, and probed with specific antibodies. Lysates are kept at 4°C until the last samples (24h) are collected. All samples from 0 to 4h from one experiment are run on one gel. The 24 h samples are run separately. Western blot densities are determined with a Licor imager and ACE-2 band densities are quantified with the FIJI variant of Image-J (27). Biotinylation densities of untreated, RBD-treated, and TIP-treated cells decrease exponentially with time. Initial densities at time 0 are set to unity and densities are normalized to the amount of biotinylated ACE-2 and values are plotted on a semi-log plot to determine rate of biotin loss.

### Electrophysiology of HL-MVEC

*Whole cell recording.* Patch electrodes are pulled on Narishige vertical puller producing electrodes which when filled with saline are approximately 10 MOhms. A giga-Ohm seal is formed on a HL-MVEC and the patch membrane is disrupted with a short pulse of current to form a whole cell patch. PClamp 10.7 is used to apply a step voltage protocol and current voltage relationships are determined. Solutions for whole-cell patch-clamping recording from HL-MVEC are in mM NaCl: 140; KCl: 5; CaCl_2_:1; MgCl_2_:1; HEPES: 10. pH=7.4. The pipette solution is NaCl: 5; KCl: 140; CaCl_2_: 3; MgCl_2_:1; EGTA: 5; HEPES: 10. pH=7.4; final Ca^2+^concentration is 100 nM.

Varying concentrations of peptide are perfused on the apical surface of the cells to a final concentration of 0, 2, 5, 10, 25, or rarely 50 μg/ml; i.e., 0, 5.2, 10.4, 26, 52, 130, or 260 nM. The holding potential is -40 mV with voltage steps from -100 to +60 in steps of 10 mV. The current at -100 mV is recorded to measure ENaC response. Amiloride (10 μM) is added at the end of the protocol to determine the component of the current due to ENaC.

*Single channel cell-attached recording.* Single channel events are recorded with an Axopatch 1D and then digitized at 4000 Hz with an Axon 1440 digitizer directly to disk storage. Patch pipettes with a resistance of 6–10 MΩ are fabricated from filamented borosilicate glass capillaries (TW-150F; World Precision Instruments) with a two-stage vertical puller (PP-2; Narishige, Tokyo, Japan). Cells are visualized with Hoffman modulation optics (Modulation Optics, New Haven, CT, USA) on a Nikon Diaphot. Negative pressure is applied to achieve a cell-attached patch with a seal resistance of greater than 10 GΩ after making contact between the pipette tip and the cell surface. The extracellular bath solution consists of a saline solution (150 mM NaCl, 5 mM KCl, 1 mM CaCl_2_, 2 mM MgCl_2_, 5 mM glucose, and 10 mM HEPES, adjusted to pH 7.4). The patch pipette solution consists of a saline solution (140 mM NaCl, 2 mM MgCl_2_, and 10 mM HEPES, adjusted to pH 7.4). The cell-attached patch configuration is used for single-channel experiments, and voltages are given as the negative of the patch pipette potential. The initial patch pipette potential is +40 mV, as such driving cations inward across the membrane. ENaC activity and open and closed times within a patch are calculated using pCLAMP 10 software (version 10.7, Molecular Devices, San Jose, CA, USA). We use the product of the number of channels (*N*) times the single channel open probability (*P_o_*) as a measure of channel activity within a patch. This product is calculated without making any assumptions about the total numbers of channels in a patch or the open probability time (*P_o_*) of a single channel. The total number of functional channels (*N*) in a patch is estimated by observing the number of peaks in the current-amplitude histogram over the entire duration of the recording, after which *P_o_* can be calculated from *NP_o_* and *N*.

### Detection of fibrin deposition in lungs in immunostaining

Lungs are flushed through the right ventricle of the heart with warm PBS to be clear of blood, fully inflated with 10% formalin via intratracheal instillation, and excised *en bloc* after trachea ligation. Lungs are subsequently fixed in 10% formalin overnight and included in paraffin. Lung sections are cut at 5 µm and mounted on glass slides. Sections are incubated in an oven at 80°C for 30 min, deparaffinized with xylene and cleaned in ethanol and water. Deparaffinized sections are placed overnight in antigen retrieval solution (10 mM sodium citrate buffer, pH= 6) at 65–80°C. Sections are then removed from the oven to room temperature and allowed to cool for about 20 min, before proceeding with immunostaining. We use a primary anti-fibrin mouse monoclonal antibody (clone 59D8, Cat. No. MABS2155, Sigma-Aldrich) and a commercial kit (M.O.M.® ImmPRESS® HRP (Peroxidase) Polymer Kit, MP-2400, Vector), that contains a mouse Ig blocking reagent paired with a specialized, ready-to-use, one-step M.O.M. ImmPRESS Peroxidase Polymer reagent. Detection of fibrin deposition is achieved using diaminobenzidine (DAB) substrate (ImmPACT® DAB Substrate Kit, Peroxidase (HRP), SK-4105, Vector), which produces a brown reaction product. Sections are counterstained with hematoxylin, dehydrated, cleared in xylene and mounted (Vecta Mount, H-5000, Vector) for microscopic analysis.

### Tissue factor detection in HL-MVEC

Human Tissue factor ELISA kit is from Abcam (Cat # Ab220653).

### Statistical analysis

All data are presented as means ± SD. Data are analyzed by GraphPad InStat software (GraphPad Software Inc.). Comparisons between groups are analyzed using one or two-way analysis of variance (ANOVA) with the Tukey’s *post hoc* test. P < 0.05 is defined as statistically significant.

## Results

### TIP peptide increases whole cell amiloride-sensitive current in HL-MVEC

**Figure 1:**
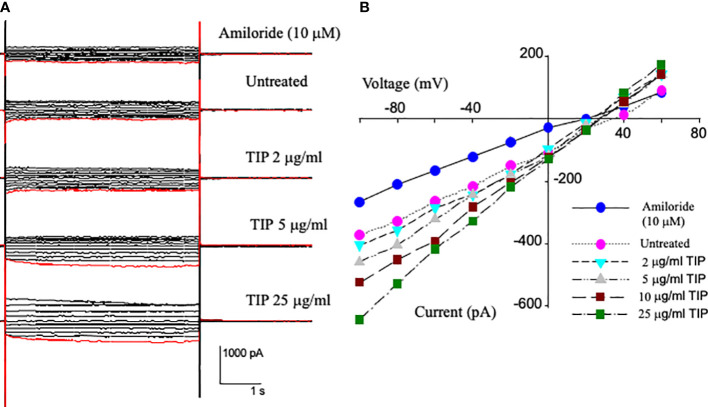
Current-Voltage relationships from whole cell patch recordings in HL-MVEC. **(A)** On the left are the current records. The holding potential was -40 mV with voltage steps from -100 to +60 in steps of 10 mV. The TIP peptide concentrations were 0, 2, 5, 10, and 25 μg/ml. 10 μM amiloride was added after the highest concentration of peptide. **(B)** current-voltage relationships at different TIP concentrations and amiloride sensitive current at -100 mV is 106 pA. The TIP-induced increase in amiloride-sensitive current implies that TIP peptide activates a sodium conductance likely ENaC. This current also implies that there are approximately 500 sodium conductive channels per cell and that TIP peptide increases their open probability.

We have previously shown that lung capillary endothelial cells express all three subunits of the epithelial sodium channel (ENaC): α, β and γ^25^). In order to investigate whether TIP peptide can actually activate amiloride-sensitive Na+ uptake, we applied voltage steps in 10 mV increments from -100 mV to +60 mV to whole cell patches on HLMVEC cells and measured the current before and after applying increasing concentrations of TIP peptide. As shown in **Figure 1B**, TIP peptide increases the cellular current at all potentials. We then added 10μM amiloride to block ENaC channels. Amiloride reduces the whole cell current to a level below that of untreated cells showing that there is significant amiloride-sensitive current before addition of TIP peptide in HLMVEC and that amiloride can also block all of the TIP-induced current. The reversal potential for the amiloride-sensitive currents were all very positive consistent with the currents being due to ENaC.

### SARS-CoV2 RBD inhibits ENaC activity in HL-MVEC

**Figure 2:**
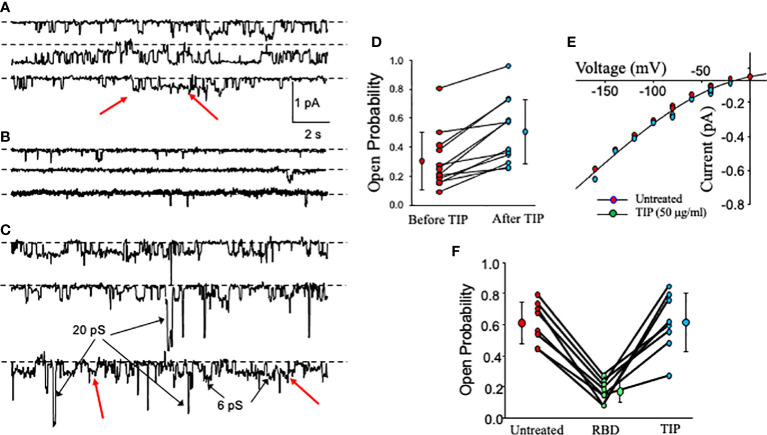
Effect of RBD, followed by TIP peptide, on activity of cation-permeable channels in HL-MVEC. **(A–C)** show continuous single channel recordings from HL-MVEC cells in culture all at a patch pipette potential of +40 mV. Recordings are from untreated cells **(A)**, cells treated with SARS-CoV2 RBD (2 μg/ml) for 5 min prior to recording **(B)**, and subsequent treatment of the same cell for 6 min with TIP peptide (50 μg/ml) (continuous record for 40 s) **(C)**. Most of the channels are ENaC channels that we have previously shown to be sensitive to less than 1 μm amiloride. Some non-selective cation channels are also present (examples are marked with arrows in **C**). Dotted lines associated with each trace mark the zero current state (all channels closed). On the right in **(D)** is the effect of TIP on ENaC channels in HL-MVEC cells. As is typical for ENaC, the open probability varies over a wide range (0.0897 to 0.806 before TIP peptide and 0.251 to 0.959 after TIP peptide). The treatments are significantly different by paired t test at p < 0.001 (before TIP 0.302 ± 0.199 compared with 0.505 ± 0.225 mean ± s.d. n=12). **(E)** shows the current voltage relationships for unit single channel currents for untreated and TIP treated patches for both conditions the current is nonlinear which is characteristic of low conductance ENaC channels. There is no significant difference between these two conditions. This implies that TIP does not alter the channel open state. **(F)** summarizes the results from 8 experiments on the effect of SARS-CoV2 S1 (RBD) on ENaC channel open probability. This graph and **(A–C)** show that the time in the open state is reduced by the S1 protein from that of untreated cell, but that channel openings are restored by addition of TIP peptide. Untreated and TIP-treated are not significantly different (Untreated and TIP open probability = 0.611 ± 0.134 and 0.613 ± 0.187 mean ± s.d.; p = 0.977). TIP and untreated vs RBD (RBD open probability = 0.168 ± 0.0705 mean ± s.d.; p <0.001; both by repeated measures ANOVA with Holm-Sidak post-test, n = 9).

We have recently shown that activation of endothelial ENaC can preserve barrier function in lung capillaries in the presence of bacterial toxins (24, 25). Previously, others have shown that the spike proteins of both SARS-CoV and SARS-CoV2 can inhibit ENaC activity in Xenopus oocytes expressing the α, β and γ subunits of human ENaC (28, 29). However, whether the RBD fragment of the spike protein S1 subunit can also reduce ENaC activity in primary HL- MVEC and whether ENaC can still be activated by TIP peptide in the presence of SARS-CoV2 RBD in these cells has not been investigated. As shown in single channel patch clamp measurements in **Figure 2A**, basal ENaC activity in HL-MVEC is strongly reduced after a 5 min treatment with recombinant SARS-CoV2 RBD (RayBiotech, 2 μg/ml, middle panel) (**Figure 2B**) and this is significantly relieved after the subsequent addition of TIP peptide (50 μg/ml) for 6 min (**Figure 2C**). **Figure 2D** shows that, as anticipated from whole cell currents, TIP peptide significantly increases ENaC open probability but without a significant change in the number of channels per patch. **Figure 2E** show the relationship between voltage and the amplitude of single channel events. Despite changing the open probability, TIP does not change the amplitude of single channel events which implies that TIP does not alter ENaC’s conducting pore. The slight curvature is characteristic of ENaC current and due to more Na^+^ charge carriers on the outside of the cell than on the inside. A summary of the effects of RBD and TIP from 8 experiments like the one shown in **Figures 2A–C** is in **Figure 2F**. RBD significantly inhibits ENaC open probability (*Po*) and that this is restored by subsequent TIP peptide addition (*: p<0.05 vs. vehicle control or TIP treated group, n=9 per group). We could detect a similar inhibitory activity with full length recombinant SARS-CoV2 spike protein (Creative Diagnostics, data not shown), confirming previous findings by others (29). Data from whole cell (**Figure 1**) and single cell patch clamp measurements (**Figure 2**) convince us that HL-MVEC express amiloride-sensitive low conductance ENaC channels and that these channels can be inhibited by RBD and that TIP peptide can restore their activity.

### Endothelial ENaC activation by TIP peptide increases surface hACE2 expression in RBD-treated HL-MVEC

**Figure 3:**
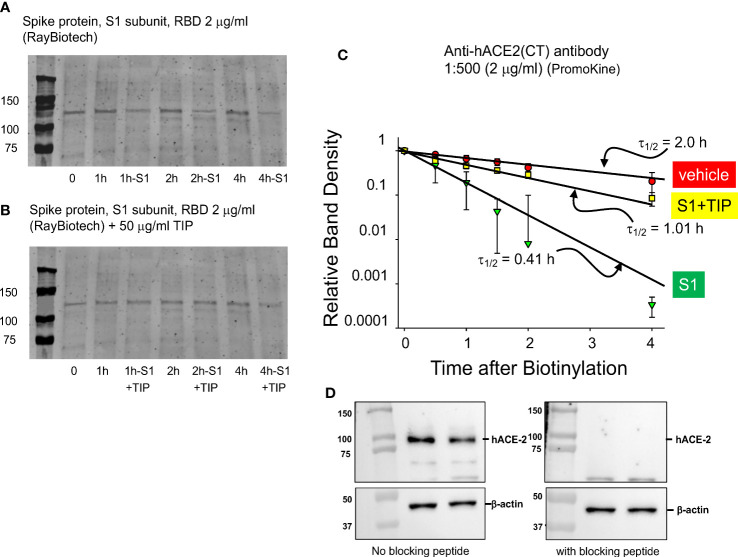
TIP peptide partially inhibits RBD-induced reduction in hACE-2 surface expression. **(A, B)** Representative surface biotinylation experiment. Glycosylated hACE-2 (i.e., the target for biotinylation) runs at about 130 kDa. We assessed hACE-2 surface expression in HL-MVEC treated with either vehicle (control group), RBD or RBD+TIP. Please note that the same control group is shown in **(A, B)**, since all samples are from the same experiment. **(C)** Results of 3 separate experiments with untreated, RBD-treated, and RBD+TIP-treated HL-MVEC. Half-life after RBD treatment implies rapid removal of hACE-2 from the membrane by shedding and/or internalization. TIP peptide partially reverses the effect of RBD. Data are normalized to the difference of biotin label at time 0 and at small amount of label remaining at 24h and then fit to a single exponential decrease. Difference in half-lives for RBD vs. vehicle or RBD+TIP-treated are significant (p < 0.001, n =3, t-test). **(D)** Specificity of the anti-hACE2 antibody, upon pre-incubation with blocking peptide (1:1 molar ratio, 1h, right panel) or not (left panel).

Human ACE-2 is the main receptor and point of entry for SARS-CoV2 in the alveolar compartment of the lungs, as such motivating strategies to reduce its expression in alveolar epithelial cells in COVID-19 (30). However, reduced hACE-2 expression in endothelial cells, as can occur through hACE-2 ectodomain shedding (31–33), will shift the ACE2/ACE balance towards generation of barrier-disruptive angiotensin 2 (Ang 2). Increased Ang 2 binding to the angiotensin type 1 receptor (AT1R) in capillary endothelial cells can trigger a signaling cascade, culminating in the shedding of additional ACE-2 and further loss of barrier function. Circulating plasma Ang 2 levels are markedly elevated in COVID-19 patients, as compared to healthy controls and correlate with the severity of lung injury (34). Consistent with data reported by other groups (33), RBD (2 μg/ml) time-dependently reduces surface hACE-2 expression in HL-MVEC. We used surface biotinylation (**Figures 3A, C**) to estimate the rate of loss of biotinylated hACE2 from the surface membrane. Loss of hACE2 surface expression is actually 5 times faster in the presence of RBD (2 μg/ml) compared to vehicle-treated cells. As demonstrated in **Figures 3B, C**, co-treatment with TIP peptide (50 μg/ml) significantly preserves hACE-2 surface expression in cells treated with RBD. **Figure 3D** documents the specificity of the antibody signal, which is abrogated upon preincubation of the antibody with the peptide immunogen against which it is was raised (1:1 molar ratio).

### TIP peptide significantly reduces RBD-induced oxidative stress in HL-MVEC

**Figure 4:**
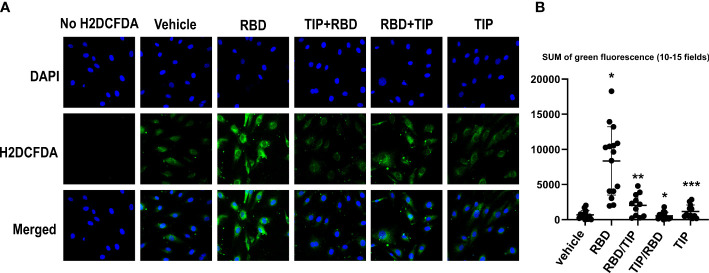
Direct ENaC activation blunts RBD-induced ROS generation in HL-MVEC. **(A)** Representative H2DCFDA staining as an indicator of ROS in HL-MVEC treated for 2h with SARS-CoV2 Spike protein RBD (5 μg/ml, RayBiotech), upon pre- (-30 min, TIP-RBD)) or post-treatment (+1h, RBD-TIP) with TIP peptide (50 μg/ml), in comparison to vehicle-treated or TIP peptide only-treated cells. **(B)** Quantification of total green fluorescence in 10-15 fields (* p<0.03: RBD vs. Vehicle, TIP-RBD vs. RBD; ** p<0.05: RBD-TIP vs. RBD and Vehicle; *** p<0.04: TIP vs. RBD).

RBD binding to hACE2 shifts the hACE2/hACE1 balance towards Ang 2 generation, the circulating levels of which are increased in severe COVID-19 patients (30–34). Ang 2 activates PKC, which in turn phosphorylates cytosolic subunits of NADPH oxidase 2 (NOX2) and fosters their recruitment to gp91^phox^ required for NOX2 activation (35). NOX2 activation, as measured by soluble NOX2-derived peptide (sNOX2-dp) was shown to be higher in COVID-19 patients *versus* controls and in severe *versus* non-severe COVID-19 (36). As shown in **Figures 4A, B**, HL-MVEC treated for 2h with SARS-CoV2 RBD (5 μg/ml) significantly increase their reactive oxygen species (ROS) generation, as compared to vehicle control, TIP peptide-pretreated (-30 min) or post-treated (+1h) cells and as measured by H_2_DFCDA staining.

### NOX2 inhibition or ENaC activation blunt RBD-induced barrier dysfunction and Tissue Factor (TF) generation in HL-MVEC

**Figure 5:**
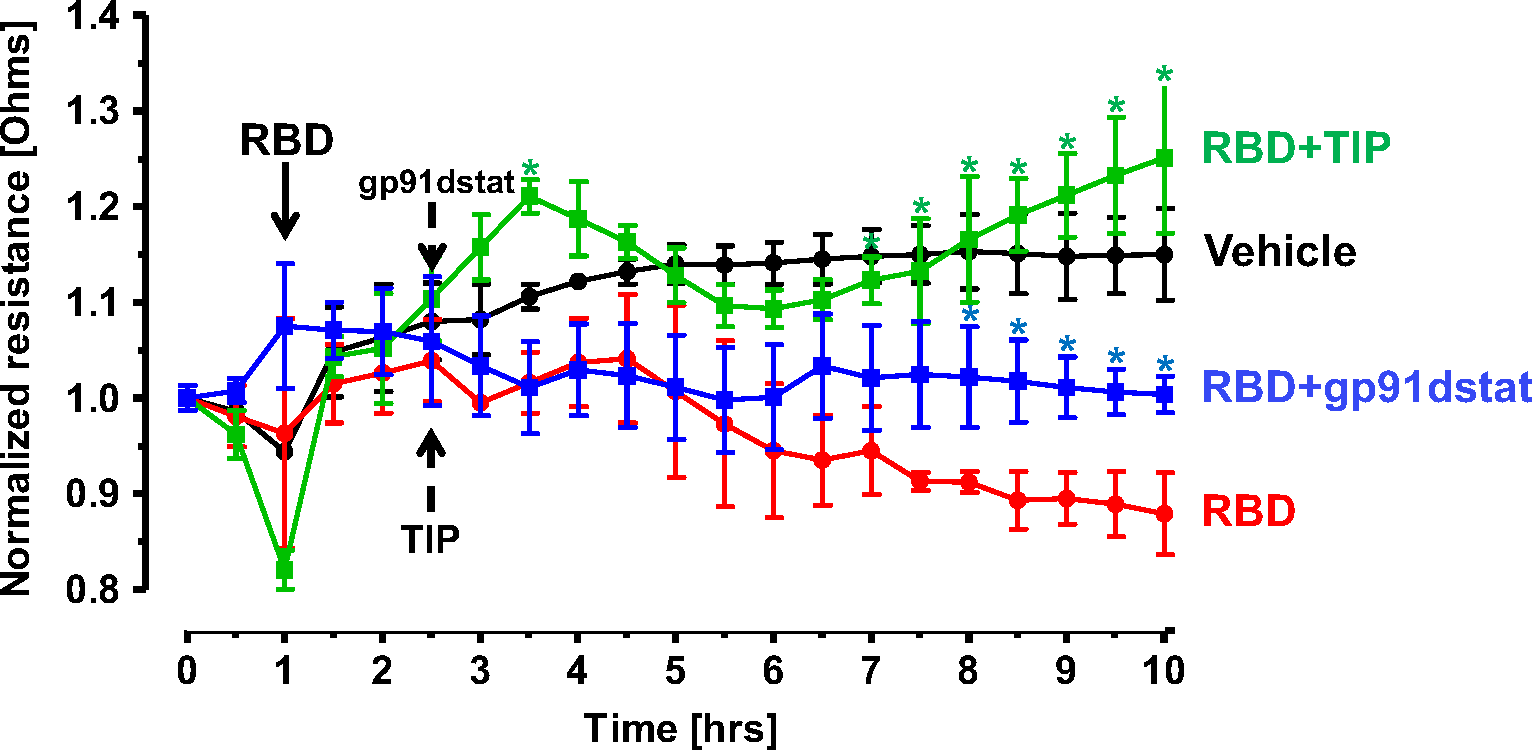
TIP peptide and NOX2 inhibitor gp91dstat partially reverse RBD-induced barrier dysfunction in HL-MVEC monolayers. TIP peptide (50 μg/ml) and NOX2 inhibitor gp91dstat (10 μM), when applied 1h after RBD, significantly restore barrier function, measured in electrical cell-substrate impedance sensing (ECIS) over the entire duration of the experiment. (n=4 per group; *:p<0.05 vs. RBD for both RBD+TIP and RBD+gp91dstat groups).

The predominant mechanisms inducing endothelial barrier impairment include, first, phosphorylation of regulatory myosin light chain (MLC) (catalyzed by either Rho kinase or Ca^2+^-dependent MLC kinase, which causes actin cytoskeleton rearrangement and formation of actin stress fibers) and, second, microtubule depolymerization, which causes disassembly of adherens junction proteins, such as vascular endothelial (VE)-cadherin (24, 37). **Figure 5** demonstrates that in accordance with findings from others, RBD (5 μg/ml) treatment induces a small, but significant increase in HL-MVEC monolayer permeability within 6h, as measured in electrical cell-substrate impedance sensing (ECIS). TIP peptide (50 μg/ml), as well as the NOX2 inhibitor gp91dstat (10 μM), significantly strengthen barrier function when applied to the cells 1h after RBD. These results indicate that oxidative stress induced by RBD is crucially involved in its barrier disruptive activity and that NOX2 activation plays a prominent role in this.

**Figure 6:**
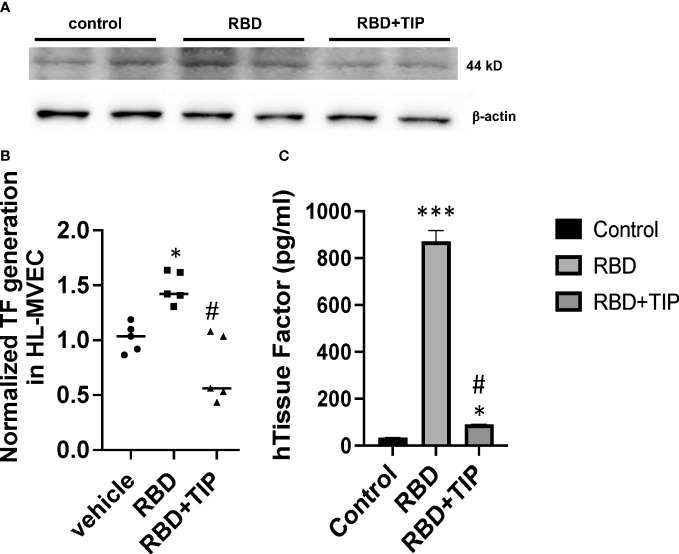
RBD increases Tissue Factor protein expression in HL-MVEC. **(A)** RBD (5 μg/ml) within 6h induces a significant increase in TF expression in HL-MVEC, which is mitigated by a 1h post-treatment with TIP peptide. **(B)** Normalized TF generation (WB) in vehicle, RBD or RBD+TIP treated HL-MVEC (n=5 per group; mean ± SD; *: p<0.0008 vs vehicle; #: p<0.0012 vs RBD). **(C)** Quantitative TF assessment (ELISA) in HL-MVEC treated for 6h with vehicle or RBD, the latter in the presence or absence of TIP peptide (50 μg/ml, 1h post RBD) (mean ± SD; n=3-5 per group; ***: p<0.0001 vs. control; #: p< 0.001 vs. RBD; *: p<0.05 vs. control).

COVID-19 ARDS has a significantly higher prevalence of microthrombi in pulmonary capillaries as compared to non-COVID ARDS (5–12). The procoagulant glycoprotein tissue factor (TF) is a major mediator of thrombosis, since it interacts with circulating coagulation factor VII to trigger extrinsic coagulation. Under conditions of severe infection and inflammation, as in severe COVID-19, TF can be released in the lungs not only by monocytes, macrophages and epithelial cells, but also by endothelial cells. TF pathway inhibitor (TFPI) -predominantly expressed by endothelial cells- may be consumed and degraded in these conditions, as e.g. in patients with sepsis (38, 39). TF generation is significantly increased in plasma and lungs from severe COVID-19 patients as compared to mild COVID-19 or control patients (8, 39, 40). Endothelial cells were recently proposed to be a critical player contributing to severe thrombosis in the lungs of COVID-19 patients (5). NF-kB-dependent induction of TF gene transcription in endothelial cells requires increased NOX activity (40), as such providing a link between endothelial oxidative stress and coagulopathy. Whether SARS-CoV2 RBD, which increases ROS generation in HL-MVEC, augments TF generation in human lung MVEC has not been investigated. Our data from a representative Western blotting study in **Figure 6A** and from densitometry from three independent experiments in **Figure 6B** demonstrate that RBD (5 μg/ml) increases TF protein generation within 6h in HL-MVEC and that this can be blunted by TIP peptide-mediated ENaC activation. Using a TF ELISA kit with a special extraction buffer, the difference between the RBD and the vehicle or RBD+TIP groups is found to be even more pronounced (**Figure 6C**).

### RBD sensitizes *S. pneumoniae*-infected hACE2 mice for pulmonary fibrin deposition and capillary leak

**Figure 7:**
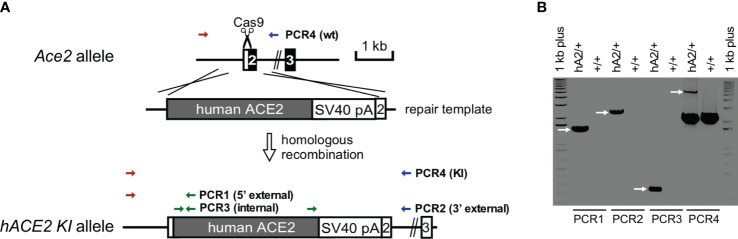
Generation and confirmation of *hACE2* knock-in founder mice. **(A)** *hACE2* knock-in targeting strategy by CRISPR/Cas9. **(B)** PCR genotyping of *hACE2* knock-in founder. PCR reactions are performed to identify the unique 1.5 kb of 5’ external, 2.2 of 3’ external, and 0.27 kb of internal products in the correctly targeted founder, respectively. Additional PCRs are used to genotype *Ace2* wild-type (2.2 kb) and *hACE2* knock-in (5.5 kb) alleles.

**Figure 8:**
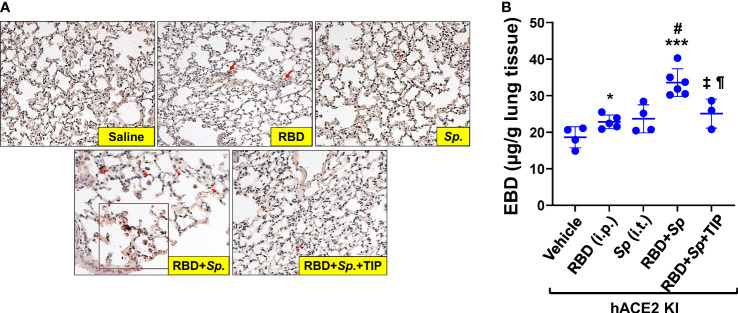
RBD sensitizes mice to *Sp*.-induced lung fibrin deposition and capillary leak. **(A)** Increased fibrin deposition in lung sections from RBD+*Sp*.-treated mice, as compared to vehicle, RBD alone or *Sp.* alone and reduction by TIP peptide treatment (applied 1h post RBD i.p.). Primary Ab: Anti-Fibrin, clone 59D8, Cat. No. MABS2155 (Sigma-Aldrich), Secondary Ab: M.O.M.® (Mouse on Mouse) ImmPRESS® HRP (Peroxidase) Polymer Kit (Vector labs). **(B)** RBD (i.p.) induces a modest increase in capillary leak by 48h in 8-10 wk old female hACE2 Knock In mice. Infection with 10^6^ CFU *S. pneumoniae* (Sp.), which by itself does not significantly increase capillary leak by 24h in these mice, induces a profound leak when combined with RBD, given 24h before *Sp*. TIP peptide (i.p. 1h post RBD) significantly mitigates the leak induced by the RBD/*Sp*. combination (Mean + SD, n=3-6 per group. *: p<0.03; ***: p<0.0002 and ¶: p<0.05 vs. Vehicle. #: p<0.004 vs. RBD; ±: p<0.02 vs. RBD+*Sp*.).

Although mice are not hosts for SARS-CoV-2, expression of human ACE-2 is sufficient, in itself, to confer susceptibility to the virus in this species (41). In order to investigate whether RBD can sensitize lung endothelium for pneumococcal pneumonia-induced ARDS and coagulopathy, we have generated transgenic mice, in which mouse ACE2 has been globally replaced with human ACE2 (global hACE-2 knock in mice), using CRISPR-Cas9 (**Figures 7A, B**). These mice also express human ACE-2 in pulmonary endothelial cells. 8-10 wk old female global hACE2 knock in mice (hACE2 KI) are injected on day 0 intraperitoneally (i, p.) with RBD of SARS-CoV2 Spike protein (500 µg/kg), followed 1h later by either TIP peptide (2.5 mg/kg) or Saline i.p. On day 1, mice receive intratracheal instillation of either a low dose of *Streptococcus pneumoniae (Sp.*, 10^6^ CFU) or saline and on day 2 animals are euthanized. In a first pool of mice, lungs are flushed intravenously with warm PBS, inflated intratracheally with 1-1.5 ml of 10% formalin and excised *en bloc* after trachea ligation. Lungs are fixed in formalin overnight and included in paraffin for histology evaluation and immunostaining for fibrin deposits. The second pool of mice is selected for capillary leak measurements following euthanasia, which is performed by intravenous infusion of Evans Blue dye, as described (24). As shown in **Figure 8A**, at 48h after the start of the study, both RBD (given i.p), and *Sp.* (i.t. instillation) induce a minor increase in fibrin deposits (red arrows) in lung tissue. However, the combination of both induces a significantly higher fibrin signal. TIP peptide treatment, 1h after RBD, significantly reduces lung fibrin deposition in the combined RBD/Sp. group (**Figure 8A**). Intraperitoneal injection of 500 μg/kg RBD induces a significant, but modest increase in capillary leak in 8-10 wk old female global hACE2 KI mice 48h post injection (**Figure 8B**). However, when RBD is combined with an i.t. instillation of a low dose of *S. pneumoniae* (10^6^ CFU, does not induce significant barrier dysfunction by itself) 24h post RBD, a profound capillary leak occurs. TIP peptide, given 1h post RBD i.p., significantly mitigates capillary leak induced by the RBD/*S. pneumoniae* combination. Taken together, these data indicate that RBD can sensitize hACE2 mice for indirect and direct barrier dysfunction in pneumococcal pneumonia.

## Discussion

Although therapeutic strategies to blunt lung damage in COVID-19 have mainly focused on the airway and alveolar epithelium -the main points of entry for the virus- COVID-19, particularly in the later complicated stages, is a disease of endothelial cells, and this is particularly prominent in the pulmonary microvasculature (30, 33, 42). Using automated quantitative CT measures and dynamic contrast-enhanced MRI, microvascular perfusion abnormalities can be detected in COVID-19 patients, even months after infection, but the main cause of these findings (microvascular thrombosis, remodeling, inflammation) remains to be determined (42–44). As such, it is highly significant to better characterize pathophysiological pathways involved in COVID-19-associated vasculopathy and to identify novel therapeutic strategies to curb endothelial dysfunction in both acute SARS-CoV-2 infection and in post-acute sequelae, as can occur in long COVID. The ACE/Angiotensin 2/PKC/NOX2 pathway, which is initiated by RBD-induced hACE2 shedding, represents a major therapeutic target in COVID-19 vasculopathy. Indeed, NOX2 is found activated in serum from severe COVID-19 patients (36) and it can activate the TF pathway in lung capillaries -which in turn fosters coagulopathy (31, 39) and barrier dysfunction (32). Although reduced surface expression of endothelial hACE2 by SARS-CoV2 RBD would be expected to decrease infectivity of the virus in capillaries and as such protect this compartment, a dysregulated renin-angiotensin system due to a disturbed ACE2/ACE balance will rather aggravate endothelial dysfunction in COVID-19 (33).

Our findings from this study suggest that SARS-CoV2 RBD -mainly by increasing oxidative stress-can sensitize lung capillaries towards barrier dysfunction and microthrombus formation, which synergizes with lung injury induced by pneumococcal infection.

HL-MVEC express the main entry receptor for SARS-CoV2, i.e. hACE-2 (33), as well as the protease furin (both in the Golgi and at the plasma membrane (2)), required for spike protein maturation. Intriguingly, SARS-CoV2 spike protein shares an eight-residue furin cleavage site only with one other mammalian protein, i. e. human ENaC-α (45), where it is necessary for maturation of the α and γ subunits (46, 47). It has been proposed that hijacking of furin by spike protein is one of the mechanisms by which SARS-CoV2 can interfere with ENaC activity (29, 45). This also questions the potential value of furin inhibitors in COVID-19, since these would not only impair spike protein but also ENaC α and γ subunit maturation. Another mechanism by which spike protein can affect ENaC activity is through PKC activation (28, 48). Several observations from our study convince us that HL-MVEC express low conductance ENaC channels: (1) we have shown that they contain amiloride sensitive currents; (2) single channel records have the low current and long open times characteristic of low conductance ENaC and (3) the current-voltage relationship has the inwardly rectifying current characteristic of a sodium channel, in particular, ENaC. Since our results in HL-MVEC show a potent inhibition of ENaC open probability (*Po*) with RBD, to the same extent as what we observed with full length recombinant spike protein (2 μg/ml, Creative Diagnostics, data not shown), this indicates that impaired furin cleavage is not the main inhibitory mechanism, at least in HL-MVEC. We demonstrate here for the first time that direct ENaC activation by the TIP peptide (a mimic of the lectin-like domain of TNF), which binds to the α subunit of the channel (49), potently restores hACE2 surface expression in RBD-treated HL-MVEC.

Although restoration of hACE2 surface expression is likely barrier-protective in the endothelial compartment, this may not be the case in alveolar epithelial cells, which also express ENaC and where hACE2 is an important point of entry for SARS-CoV2. As such, if TIP peptide would increase surface expression of hACE2 in alveolar epithelial cells, that could increase infectivity. It should however also be noted that -on the positive side- ENaC activation in type I and II alveolar epithelial cells, as occurs with TIP peptide, can improve vectorial Na^+^ transport and subsequent alveolar fluid clearance (AFC) (50, 51). AFC capacity was shown to inversely correlate with mortality in ARDS patients (52) and TIP peptide was shown to significantly reduce extravascular lung water in a sub-group of ARDS patients with a SOFA score >11 in a double-blind phase 2a clinical trial (53). Currently, a dose-escalating multi-center phase 2 clinical trial with TIP peptide in both non-COVID and COVID ARDS patients is underway (54). During COVID-19, apart from ENaC, also the Na^+^-K-ATPase, another crucial mediator of vectorial Na+ transport, can be impaired (55). Although TIP peptide can override the inhibitory effect of RBD on ENaC it remains to be evaluated whether it can also restore activity of the Na+-K+-ATPase. In conclusion, further studies are necessary to investigate the outcome of ENaC activation in the alveolar epithelial compartment in COVID-19 (56).

Imbalance of the hACE2/hACE activity ratio promotes Ang 2 generation and subsequent PKC activation. PKC in turn phosphorylates cytosolic NOX subunits and recruits them to the plasma membrane for full enzymatic activation. NOX activation has also been proposed as a major driver of long COVID, thus suggesting that anti-oxidative agents should be further studied as therapeutic candidates (57). Our data show that RBD potently induces oxidative stress in HL-MVEC and that ENaC activation can override this, even when applied 1h after RBD, although the mechanisms involved remain unclear. In our study, especially NOX2 seems to be an important mediator of RBD-induced barrier dysfunction, in view of the neutralizing effect of the specific NOX2 peptide inhibitor gp91dstat. Global NOX2 inhibition however does not seem to be a plausible strategy in COVID-19, since it would also abrogate the enzyme’s activity in phagocytes, crucial for host defense to infections (58). Whether the TIP peptide will also blunt NOX2 activity in phagocytes during ARDS remains to be investigated.

Our study has several limitations. First, we obviously cannot fully reproduce COVID-19 endotheliitis in the absence of live SARS-CoV2. Second, although spike protein mediates a lot of actions of SARS-CoV2 in mammalian cells, it does not account for all of them. Indeed, apart from the spike protein, also the envelope protein (E) of SARS-CoV and SARS-CoV2 has been shown to impair ENaC activity28,29. Third, apart from viral components, virally-induced host factors can also contribute to barrier dysfunction. Here it is intriguing to note that no correlation can be found between COVID-19 plasma levels of pro-inflammatory cytokines and chemokines, such as TNF, IL-6 and IL-8 and barrier disruption in HL-MVEC in ECIS20.

Our data also show that RBD by itself does not induce a strong capillary leak in global hACE2 KI mice. At first glance, this result may look different from the strong barrier-disruptive effects reported in a recent study by others (18). However, there are important differences between the two studies. First, these researchers used a different mouse model, *i.e.* K18-hACE2 mice (do not express hACE2 in endothelium) and second, they used a different route of application (i.t.), which mainly targets the airway epithelium. Intraperitoneal RBD treatment in our studies sensitizes lung capillaries in hACE2 KI mice towards coagulopathy and barrier dysfunction during mild pneumococcal pneumonia. This finding might be relevant to the significantly increased mortality in COVID patients co-infected with *S. pneumoniae* (14).

In conclusion, our study demonstrates that RBD can sensitize both hACE2 KI mouse and human lung capillaries towards coagulopathy and barrier dysfunction, by a mechanism involving impairment of endothelial ENaC, shedding of hACE2 and activation of NOX2. Since plasma RBD levels correlate with disease severity in COVI-19, these results can at least partially provide an explanation for the higher vasculopathy observed in COVID-ARDS, as compared to non-COVID ARDS. Direct activation of endothelial ENaC can override these RBD actions in lung capillaries and should be further evaluated as a potential therapeutic strategy to curb SARS-CoV2-induced vasculopathy and long COVID.

## Data availability statement

The raw data supporting the conclusions of this article will be made available by the authors, without undue reservation.

## Ethics statement

Ethical approval was not required for the studies on humans in accordance with the local legislation and institutional requirements because only commercially available established cell lines were used. The animal study was approved by the Institutional Animal Care and Use Committee at Augusta University. The study was conducted in accordance with the local legislation and institutional requirements.

## Author contributions

Conceptualization: MR, BA, TC, GC, DE, RL. Experimental and data analyses: MR, QY, BS, SS, MAM, CS, DF, LG, GC, DE, RL. Project Administration: DE, RL. Supervision: DE, RL. Original draft preparation: MR, QY, JH, TC, DE, RL. Reviewing and Editing: MR, JH, BF, MZ, MM, DF, GC, DE, RL. All authors contributed to the article and approved the submitted version.
